# Antiviral Surface Coatings: From Pandemic Lessons to Visible-Light-Activated Films

**DOI:** 10.3390/ma18040906

**Published:** 2025-02-19

**Authors:** Plinio Innocenzi

**Affiliations:** Laboratory of Materials Science and Nanotechnology, Consorzio Interuniversitario per la Scienza e Tecnologia dei Materiali (INSTM), Department of Biomedical Sciences, University of Sassari, Viale San Pietro 43/B, 07100 Sassari, Italy; plinio@uniss.it

**Keywords:** antiviral, coatings, reactive oxygen species, fomite, biocidal surfaces

## Abstract

The increasing need for effective antiviral strategies has led to the development of innovative surface coatings to combat the transmission of viruses via fomites. The aim of this review is to critically assess the efficacy of antiviral coatings in mitigating virus transmission, particularly those activated by visible light. The alarm created by the COVID-19 pandemic, including the initial uncertainty about the mechanisms of its spread, attracted attention to fomites as a possible source of virus transmission. However, later research has shown that surface-dependent infection mechanisms need to be carefully evaluated experimentally. By briefly analyzing virus–surface interactions and their implications, this review highlights the importance of shifting to innovative solutions. In particular, visible-light-activated antiviral coatings that use reactive oxygen species such as singlet oxygen to disrupt viral components have emerged as promising options. These coatings can allow for obtaining safe, continuous, and long-term active biocidal surfaces suitable for various applications, including healthcare environments and public spaces. This review indicates that while the significance of fomite transmission is context-dependent, advances in material science provide actionable pathways for designing multifunctional, visible-light-activated antiviral coatings. These innovations align with the lessons learned from the COVID-19 pandemic and pave the way for sustainable, broad-spectrum antiviral solutions capable of addressing future public health challenges.

## 1. Introduction

Common objects with which we frequently come into contact during our daily lives can turn into fomites and become the hosts of infectious agents, such as bacteria, viruses, or fungi, potentially facilitating their transmission from one individual to another [1]. Fomites are defined as objects in the environment on which infectious agents can survive, becoming a potential vector for person-to-person transmission. These materials’ surfaces are not inherently infectious, and they become fomites only when contaminated with pathogens. Fomites serve as indirect vehicles for infections, enabling the spread of diseases when individuals touch contaminated objects and then their own mucous membranes, e.g., eyes, nose, mouth, etc. [2]. Common examples of fomites include the surfaces of door handles, mobile phones, toys, utensils, bed linen, and medical equipment such as stethoscopes and thermometers. Because in our daily lives, we continuously touch surfaces, much care should be taken in the choice of materials in critical environments for health, such as schools, long-term care facilities, surgery and emergency rooms, and hospitals in general. Such surfaces are defined as “high-touch surfaces”, i.e., those surfaces that are frequently “touched” by the hands and represent “the surfaces” most frequently contaminated by microorganisms [3,4].

The survival of viruses and bacteria on fomites depends on many factors, some external, such as temperature and humidity, and others inherently dependent on the type of material and the surface properties, such as porosity and wettability. For fomite transmission to occur, the virus must be transferred from the surface to the mucous membranes (eyes, nose, or mouth) in a sufficient dose to establish infection [5]. Evidence suggests that this route is less efficient compared to respiratory droplet or aerosol transmission [6].

The types of diseases and infections that can potentially be transmitted through fomites include several common respiratory and enteric illnesses. The common viral infectious diseases that can potentially spread via fomites include the following: adenoviruses, a group of viruses that are the sources of infections in the upper respiratory tract and the eyes [7]; coronaviruses, such as SARS and MERS, which cause upper respiratory infections in both animals and humans [8,9,10,11]; hand foot and mouth disease, which is an infection that gives rise to fever and blisters on the hands, feet, and inside the mouth [12]; norovirus, responsible for gastroenteritis diseases [13,14,15,16]; rhinovirus, the most common viral infectious agents in humans [17,18]; and rotavirus, which is the leading cause of diarrhea in infants and young children [19].

Compared with previous reviews, this article incorporates some insights and lessons learned from the COVID-19 pandemic. It critically evaluates the actual significance of fomite transmission during the pandemic and its implications for future surface designs. Regarding antiviral surface technologies, particular attention is dedicated to the unique potential of visible-light-activated coatings. These coatings offer long-term disinfection via safe, ambient light, contrasting with traditional UV or chemical-based methods. This review includes comparative tables summarizing the strengths, challenges, and applications of various antiviral coating technologies, such as porphyrin-based, carbon-based, and nanoparticle-enhanced systems. These practical insights provide a first general understanding of the challenges still posed in understanding and controlling viral infections from fomites.

## 2. Viral Infection from Fomites, Narrative or a Scientific Case?

The outbreak of COVID-19 from SARS-CoV-2 has drawn attention to fomites as a possible means of spreading the infection. The COVID-19 pandemic has promoted, for the first time, a series of systematic studies on the effects of fomites in the diffusion of viral infections [20,21]. In the early phase of the pandemic, the alarm created by the possibility of infection through surfaces led to the indiscriminate use of disinfectant agents that later proved unnecessary in retrospect.

Early studies on COVID-19 infection showed that the virus in the laboratory can survive on different types of surfaces, such as plastic, stainless steel, and cardboard, for periods ranging from 4 to 72 h [22,23,24]. However, later research has provided a more nuanced understanding, and it has been demonstrated that the transmission of infection through fomites is a very rare, if not impossible, event [25,26,27,28,29].

Interpretation of these results is also not easy due to a lack of protocols, making comparative evaluation of these studies unfeasible [30,31]. Unlike droplet or airborne transmission, proving fomite transmission involves tracing a direct link between a contaminated surface and infection, which is complex and often indirect. On the other hand, laboratory conditions do not replicate real-world environments, where desiccation, UV exposure, and cleaning practices reduce the virus’ viability. Therefore, the assessment of infection should be performed with care, even if environmental sampling in healthcare settings and public spaces identifies SARS-CoV-2 RNA on frequently touched surfaces [32]. The detection of RNA or even viable viruses on surfaces suggests that contaminated fomites may harbor viral material; however, this detection does not confirm that the virus remains infectious in real-world conditions. Furthermore, it should be taken into account that environmental factors like temperature, humidity, and UV light degrade viral particles over time [33,34]. Contact tracing and epidemiological studies [35] have found that most COVID-19 transmission events are associated with close person-to-person contact or shared airspace, rather than surface contact [36].

One interesting example is cash, which at the beginning of the COVID-19 pandemic was considered a possible medium acting as a fomite for spreading the infection [37]. However, a risk assessment evaluation demonstrated that the risk of contracting COVID-19 via person-to-person cash transactions is lower than once per 39,000 days, or 107 years, for a single person [38]. 

Current understanding places fomite transmission as a secondary route, with respiratory transmission being dominant, and epidemiological evidence suggests that respiratory droplets and aerosols are the primary modes by which SARS-CoV-2 has spread [39]. COVID-19 infects people when an infected person exhales droplets containing the virus. Other individuals can breathe in these droplets or have them land on their eyes, nose, or mouth [40]. Additionally, these droplets may contaminate surfaces where they fall. Vaccination, handwashing, social distancing, and wearing masks help reduce the risk of infection [34,41,42].

The contribution of fomites is considered relatively minor. However, some context-specific risks must be considered, and high-contact, shared surfaces in healthcare settings or public spaces may still pose a greater risk, particularly when combined with poor hand hygiene practices [43,44]. For this reason, innovations such as light-activated coatings and metal-based antiviral surfaces (e.g., copper and silver nanoparticles) [45] that can provide passive protection by reducing surface contamination are still a hot research topic.

This brief review is devoted to a general analysis of some aspects related to implementing antiviral coatings. The experience accumulated during the COVID-19 pandemic showed that a critical approach is needed in evaluating the spread of infections from surfaces. The advances in research on this topic have allowed for a more realistic view of the effect of fomites while at the same time opening new perspectives in understanding the interaction of viruses and bacteria with surfaces. The creation of surfaces with effective antiviral properties and the field of application of such surfaces, in light of the COVID-19 experience, will have to be reduced to particular cases and specific types of viruses that can lead to infection via fomites. For this reason, this review focuses on a concise comparative analysis of some critical parameters for antiviral surface design, e.g., the different responses between enveloped and non-enveloped viruses and surface properties. The second part of this article describes a particular case that is a harbinger of important expectations, namely that of antiviral surfaces that are photoactivated by visible light. In this case, their activity is carried out through the emission of reactive oxygen species, particularly singlet oxygen, capable of destroying or interfering with virus replication.

## 3. The Virus Matter—The Difference Between Enveloped and Non-Enveloped Viruses

A key consideration in designing an antiviral surface is that it should act as a biocide with broad-spectrum activity against viruses and bacteria. However, environmental resistance varies significantly between enveloped and non-enveloped viruses due to differences in their structural composition (Table 1) [46]. These differences influence their survival on surfaces, resistance to disinfectants, and vulnerability to environmental factors like temperature [47], humidity, and UV light [33].

Enveloped viruses, such as coronaviruses, possess a protective lipid bilayer envelope surrounding the protein capsid and nucleic acid. The lipid envelope contains glycoproteins essential for cell entry, but it is sensitive to environmental conditions and easy to disrupt. Most of the epidemics and pandemics observed in recent years, such as SARS-CoV-2, Zika, and MERS, as well as the influenza virus and herpes simplex virus, are due to enveloped viruses.

Non-enveloped viruses lack a lipid envelope and are composed of a protein capsid that encases the nucleic acid. The protein capsid is more stable and resistant to environmental stressors. Some examples include the norovirus, poliovirus, and rhinovirus.

Enveloped and non-enveloped viruses also have different responses to disinfectants [48]. In enveloped viruses, the lipid envelope makes them vulnerable to detergents, alcohols, and lipid-disrupting agents. In the absence of a lipid envelope, non-enveloped viruses are more resistant to lipid-dissolving agents. More aggressive treatments, such as bleach (sodium hypochlorite) or oxidizing agents, are necessary. For instance, norovirus resists alcohol-based sanitizers but is inactivated by chlorine-based disinfectants.

In comparison, enveloped viruses survive for shorter durations on surfaces due to their sensitivity to desiccation and environmental factors [46]. SARS-CoV-2 survives for up to 72 h on plastic, but much less on porous materials like paper [47]. Non-enveloped viruses can survive for weeks to months on surfaces, particularly in dry, low-humidity environments. Norovirus can persist on surfaces for up to two weeks.

For these reasons, particular attention should be given to antiviral surfaces against non-enveloped viruses. These are a source of greater concern in environments like hospitals and cruise ships, where outbreaks of hardy viruses like norovirus can occur. In general, enveloped viruses are easier to inactivate with detergents and alcohols, whereas non-enveloped viruses are highly resistant, requiring stronger disinfectants and more stringent control measures.

## 4. How Surface Properties Influence Virion Survival

The properties of surfaces play a key role in determining interactions with viruses and bacteria. These properties include roughness, porosity, hydrophobicity, and surface energy, which can govern the inactivation of viruses or prevent their adhesion to surfaces (Table 2) [49,50]. Particular attention must be dedicated to the design of surface properties in antiviral coatings [51,52]. A deeper understanding of the chemical–physical interactions of viruses with the interface [53] clearly needs to be further developed [54,55]. Electrostatic secondary bonding, such as van der Waals interaction, the hydrophobic effect, and hydrogen bonding play a fundamental role at the interface. Modeling such interactions requires a case-by-case approach as a function of the surfaces and viruses.

Surface roughness is a parameter that has a direct influence on surface–virus interactions. An increase in surface roughness results in a higher number of contact points between the surface and the virus. This can translate into better antiviral activity of the system. However, surface roughness must be controlled to prevent antiviral particles from nesting in the grooves of the surface, making disinfection more difficult. Smooth surfaces are often preferred in healthcare settings for easier cleaning and reduced contamination.

Surface energy influences wettability and the adhesion of viruses [56]. In general, low-energy surfaces, such as fluoropolymers, resist protein and viral attachment, while high-energy surfaces promote stronger interactions with antiviral agents or coatings. Surfaces can act as active elements, for example by producing reactive oxygen species that attack the structure of viruses, or passive elements, where controlling the surface energy can improve antiviral efficacy.

Porosity is another parameter that can be used in the design of antiviral surfaces [57]. For example, pores can act as reservoirs for antiviral agents, allowing sustained release over time [58]. Porosity allows for the deeper penetration of viruses, which might enhance interaction with antiviral agents embedded within the material. On the other hand, surfaces with high roughness or porosity may degrade faster or become harder to clean over time. Furthermore, fabricating surfaces with precise control over roughness, porosity, or surface energy can be challenging and costly.

The adhesion of viruses to surfaces is controlled by another important parameter, which is the degree of hydrophobicity of the surface [59]. Hydrophobic surfaces tend to repel water, and this reduces the possible adhesion of aerosol particles containing viruses. On the other hand, hydrophobic surfaces can also facilitate virus adhesion through non-polar interactions with proteins or the virus envelope.

Superhydrophobic surfaces minimize viral attachment by providing self-cleaning effects, where water droplets roll off and remove adhered particles [60].

These surface properties often work synergistically or antagonistically, depending on the material and application. For instance, combining surface roughness with hydrophobicity creates superhydrophobic surfaces, reducing viral adhesion and enhancing self-cleaning properties. Meanwhile, high porosity with low surface energy reduces viral adherence while allowing the incorporation of antiviral agents.

## 5. Antiviral Coatings, Different Materials and Strategies

The choice of strategy to fabricate antiviral coatings depends on the intended application, cost, and required durability [61]. Combining multiple approaches, such as nanomaterial-enhanced polymer coatings, is an emerging trend to enhance efficacy and broaden applicability. Further research into safe, scalable, and multifunctional coatings is critical to addressing future viral outbreaks. Several materials and processing technologies have been developed so far to obtain antiviral coatings. One of the main issues to be addressed is the ability of the coating to be deposited on different surfaces. The deposition of a film on a metal, glass, or polymer surface requires specific methodologies, and not all of them are flexible enough to allow universal use. In this article, the different antiviral coatings that have been developed so far are not discussed in detail. The reader can refer to several extensive reviews on this subject [62,63,64,65]. A short summary of the main advantages and drawbacks of the different types of antiviral coatings studied so far is reported in Table 3.

### 5.1. Antiviral Coatings Activated by Visible Light

Visible-light-activated antiviral coatings provide a practical and safe method for reducing viral transmission in various settings, making them an essential tool in public health and infection control strategies.

Antiviral coatings activated by visible light represent an advanced strategy for continuous disinfection. These coatings use photosensitive materials or catalysts that absorb visible light (wavelengths 400–700 nm) to generate reactive species capable of inactivating viruses, including enveloped ones like SARS-CoV-2.

Visible light excites photosensitizers or photocatalysts in the coating, transferring energy to the surrounding oxygen molecules to produce ROS, such as singlet oxygen (^1^O_2_), superoxide anions (O_2_^−^), and hydroxyl radicals (^•^OH). These ROS attack viral proteins, lipids, and nucleic acids, disrupting the viral envelope, capsid, and genome.

Furthermore, some coatings modify the surface charge under visible light. In some cases, the interaction between viral particles and the coating promotes biocidal activity. Photosensitizers on the coating directly interact with light to transfer energy to viral components, destabilizing their structure.

The main advantage of visible light activation is that unlike UV-C, visible light is safe for human exposure, allowing for continuous antiviral activity in confined environments. This opens the route for applications in specific places such as surgery rooms and space stations [66]. An intrinsic advantage is that using ambient light reduces the need for UV light sources that can be harmful upon prolonged exposure. Photoactivated coatings can have broad-spectrum activity and are effective against a wide range of viruses, including both enveloped and non-enveloped viruses, as well as bacteria. Antiviral surfaces, as opposed to disinfectants such as alcohol, whose effectiveness is exhausted almost immediately due to evaporation, have long-term effectiveness and can maintain their antiviral properties over extended periods. However, there are several drawbacks that need to be considered. One is that the effectiveness of the antiviral effect depends on the intensity of the light applied. The duration and intensity of the visible light itself are, therefore, parameters that directly influence the system’s response. At the same time, the system does not work in the dark, and this can also be a limitation in some circumstances. These limitations can be overcome by using a hybrid approach to the design of antiviral surfaces by combining photoactivation with other properties. Finally, cost and scalability represent further issues to be considered for the transfer of this technology from the laboratory to industry, as advanced materials and complex processing techniques may increase production costs.

Some examples of nanomaterials capable of producing singlet oxygen (^1^O_2_) under visible light illumination include photosensitizers and catalysts that absorb visible light and transfer energy to molecular oxygen (O_2_) to generate ^1^O_2_ [67]. These materials are employed not only as antiviral–antimicrobial coatings and in environmental disinfection but also in applications such as photodynamic therapy. The systems capable of generating singlet oxygen include molecules, oxide and metal nanoparticles, and carbon-based nanostructures, such as fullerenes, carbon dots, and carbon nanotubes [68].

Molecular-based nanosystems are generally based on porphyrins and phthalocyanines [69]. Porphyrins are organic molecules with strong absorption in the visible light range, particularly in the Soret band (400–450 nm). Phthalocyanines are macrocyclic compounds with strong visible light absorption and high efficiency in generating singlet oxygen. Other molecules used to generate singlet oxygen include organic dyes like methylene blue and rose bengal. These are well-known singlet oxygen generators, and their incorporation into nanostructures enhances their stability and applicability.

Fullerenes and their derivatives are carbon-based nanostructures that can generate singlet oxygen under visible light due to their unique electronic properties [70,71,72]. Such properties have also been observed in carbon dots [73] and carbon nanotubes. Graphene derivatives, such as graphene oxide functionalized with porphyrins or phthalocyanines, have also been developed to enhance singlet oxygen production and photosensitizers under visible light.

Semiconductor nanomaterials are one of the most common choices for antiviral surfaces. Visible-light-responsive semiconductors can generate reactive oxygen species, including singlet oxygen, through energy transfer [74]. Zinc oxide (ZnO) [75,76] and titanium dioxide (TiO_2_) [77] are doped with elements like nitrogen [78], silver [79], and copper to extend their light absorption to the visible range. An alternative is represented by bismuth-based semiconductors [80,81], such as bismuth vanadate (BiVO_4_) and bismuth oxyhalides (BiOX).

In general, molecular systems, such as porphyrins, are very efficient generators of singlet oxygen but are not very photostable. Carbon dots represent an interesting alternative, along with oxide semiconductors that need a specific design to absorb light in the visible range. Table 4 and Table 5 provide general guidelines that may aid in the design of antiviral coatings, particularly those activated by visible light. Table 4 specifically lists the various systems suitable for creating visible-light-activated antiviral coatings.

Table 5 is dedicated to listing some guidelines for the design of visible-light-activated antiviral coatings. The data in these tables slightly overlap with each other and should be used to address specific issues.

### 5.2. Effect of Reactive Oxygen Species

The resistance of enveloped and non-enveloped viruses to reactive oxygen species (ROS) is primarily determined by their structural composition. ROS, such as hydroxyl radicals (^•^OH), superoxide anions (O_2_^−^), hydrogen peroxide (H_2_O_2_), and singlet oxygen [82], attack viral components, including lipids, proteins, and nucleic acids, leading to inactivation. However, enveloped and non-enveloped viruses exhibit different levels of vulnerability to ROS due to the differences in their protective structures, as we have previously described.

The lipid bilayer envelope of enveloped viruses is sensitive to oxidative stress. This envelope contains glycoproteins whose disruption inhibits the ability of the virus to attach to the host cell and enter into it. The lipid envelope is highly vulnerable to ROS-induced peroxidation, which disrupts the viral membrane, rendering the virus non-infectious. Oxidative damage to glycoproteins and other envelope proteins further compromises the virus’ ability to infect host cells. Furthermore, ROS can directly damage the viral RNA or DNA inside the envelope, but the primary target is the lipid envelope. Enveloped viruses, such as SARS-CoV-2 and influenza, are efficiently inactivated by ROS-generating agents, such as hydrogen peroxide and photocatalytic materials.

In non-enveloped viruses, the protection is given by a protein capsid instead of a lipid envelope. This capsid is composed of tightly packed, stable proteins designed to shield the viral nucleic acid. ROS must be able to disrupt the protein barrier of the virus in order to affect its genome. The protein capsid provides significant protection against ROS, reducing the likelihood of direct damage to the nucleic acid. This requires high concentrations of ROS or prolonged exposure to ROS. Viral capsid proteins provide protection against ROS, thereby reducing direct damage to the nucleic acid. It is, therefore, the ROS that can denature the capsid proteins, exposing the nucleic acid to oxidative damage. Non-enveloped viruses, such as norovirus and poliovirus, are more resilient to ROS than enveloped viruses and require higher doses or longer exposure to ROS-producing agents for effective inactivation.

In general, ROS attack viruses through multiple mechanisms. Lipid Peroxidation (Enveloped Viruses): ROS react with unsaturated lipids in the viral envelope, disrupting membrane integrity. Protein Oxidation (All Viruses): ROS oxidize amino acid side chains, leading to protein misfolding, denaturation, and loss of function. Nucleic Acid Damage (All Viruses): ROS induce strand breaks, base modifications, and crosslinking in viral RNA or DNA, compromising replication.

An important question to address is the different antiviral actions of radicals, such as hydroxyl radicals (^•^OH), superoxide anions (O_2_^−^), and hydrogen peroxide (H_2_O_2_) (Table 6), with respect to singlet oxygen [83]. They have distinct chemical properties and mechanisms of action, leading to different antiviral efficacies. Both are effective oxidant agents, but their antiviral efficacy depends on factors like reactivity, selectivity, and stability.

Hydroxyl radicals (^•^OH) are extremely reactive and non-selective [84]. Very short-lived in biological systems, with a half-life of nanoseconds, they are generated via Fenton reactions, the photolysis of H_2_O_2_, or photocatalysis, for instance by TiO_2_ under UV illumination. They are highly effective against both enveloped and non-enveloped viruses due to their ability to damage multiple viral structures and are particularly effective in inactivating enveloped viruses by destroying the lipid bilayer. The higher oxidation power of hydroxyl radicals than singlet oxygen results in their higher antimicrobial activity. Their efficacy is limited by their short diffusion distance, requiring close proximity to the virus for activity. Meanwhile, their high reactivity also limits their stability and action radius.

Singlet oxygen (^1^O_2_) is less reactive than ^•^OH but more selective [85]. Singlet oxygen is longer-lived than hydroxyl radicals [86], with a half-life in microseconds in biological systems. It is generated via energy transfer from a photosensitizer (e.g., porphyrins, phthalocyanines) under light activation and a great advantage is that it can be produced by illumination with visible light.

In general, it is chemically reactive towards C=C double bonds. It primarily oxidizes specific targets, such as unsaturated fatty acids, leading to membrane destabilization, or sulfur-containing amino acids (e.g., cysteine, methionine) and aromatic residues, impairing viral proteins functionality. It can also cause oxidative damage to guanine bases, interfering with viral replication. Singlet oxygen is highly effective against enveloped viruses due to lipid oxidation. On the other hand, it has only moderate efficacy against non-enveloped viruses; it is thus less likely to penetrate the capsid and damage internal components. It is more selective than ^•^OH, reducing collateral damage to non-viral components. Compared to hydroxyl radicals, it has also the advantage of acting over longer distances due to its relative stability. It is therefore an ideal candidate for use in photodynamic therapies and visible-light-activated antiviral coatings.

## 6. Conclusions

The lessons learned from the COVID-19 pandemic must be carefully considered for the development of antiviral coatings. The cost-effectiveness and real effect on limiting viral infections are critical aspects that should be part of the choice of developing and using antiviral coatings in an extensive way. In some specific restricted environments, such as emergency and surgery rooms, the application of biocidal surfaces still appears viable. Biocidal surfaces should have the broadest possible activity against different types of virus and bacteria. For this purpose, the surface design must consider a combination of different properties. These properties must be carefully engineered to balance a broad and effective antiviral activity with practical challenges like durability and scalability.

Visible-light-activated coatings represent a promising innovation, offering safe and continuous disinfection through the generation of reactive oxygen species. These coatings effectively target viral components, with singlet oxygen showing particular efficacy against enveloped viruses. While hydroxyl radicals exhibit higher reactivity and broad-spectrum activity, singlet oxygen stability and specificity make it ideal for long-term applications in well-lit environments. There are several challenges that still need to be addressed. One of these is the effectiveness of action in dark and low-light conditions, which can be addressed by combining light-activated coatings with other antiviral strategies to ensure efficacy even when light levels are low. Another issue is the high costs and complexity associated with synthesizing advanced materials. The coating process should be as simple, scalable, and cost-effective as possible. Additionally, it is important to consider the environmental impact and ensure the biocompatibility of nanomaterials. Future research should focus on optimizing the interaction between material properties, viruses, and ROS generation while exploring new strategies to overcome limitations in different environmental settings.

## Figures and Tables

**Table 1:** The effect of external factors for enveloped and non-enveloped viruses.

External Factor	Enveloped Virus	Non-Enveloped Virus
**Desiccation**	Sensitive to drying due to dependence on a hydrated lipid envelope	More resistant to drying; capsid structure retains integrity
**Temperature**	Moderate resistance; extreme heat denatures envelope proteins.	Higher resistance; capsids withstand a wider range of temperatures
**Humidity**	Stability decreases at low humidity due to lipid degradation	Stability often increases at low humidity
**pH**	Sensitive to extreme pH changes that disrupt the lipid envelope	Stable across a broad pH range, including acidic and alkaline conditions
**UV Radiation**	Moderately resistant; UV can damage the viral RNA/DNA	More resistant due to protective protein capsid.
**Reactive oxygen species**	Weakly resistant, ROS disrupt the lipidic bonds	Resilient to ROS attacks

**Table 2:** Effects of surface properties on antiviral activity.

Surface Property	Effect on Antiviral Activity	Examples and Applications	Challenges
**Roughness**	Increased surface area enhances contact with antiviral agents.	Rough surfaces coated with metals like copper improve viral inactivation.	Can harbor viral particles in crevices, making cleaning difficult.
	Too much roughness may shield viruses from external disinfectants.	Antiviral coatings for high-touch surfaces like door handles.	Requires precise engineering to balance efficacy and cleanliness
**Porosity**	Porous surfaces act as reservoirs for sustained release of antiviral agents.	Metal–organic frameworks loaded with silver or copper for continuous antiviral action.	Excessive porosity can trap viruses, reducing efficacy of surface cleaning.
	Promotes interaction between viruses and embedded antiviral agents.	Porous membranes used in air and water filters and self-sterilizing materials.	May reduce mechanical strength of materials in some applications.
**Hydrophobicity**	Hydrophobic surfaces repel waterborne viruses, reducing adhesion.	Superhydrophobic surfaces prevent viral contamination via self-cleaning properties.	Non-polar interactions may enhance adhesion of some enveloped viruses.
	Creates self-cleaning surfaces where water droplets roll off, removing contaminants.	Commonly applied in medical textiles, PPE, and protective coatings.	Requires robust materials to maintain hydrophobicity over time.
**Hydrophilicity**	Increases viral interaction by enhancing surface wettability.	Hydrophilic coatings with embedded ROS generators improve inactivation efficiency.	Excessive water retention may reduce long-term antiviral efficacy.
**Surface Energy**	High surface energy promotes strong adhesion of antiviral agents or coatings.	High-energy surfaces, such as titania, enhance ROS production for photodynamic antiviral activity.	Can increase adherence of contaminants if not combined with effective antiviral coatings.
	Low-energy surfaces resist viral adhesion, reducing contamination risk.	Low-energy fluoropolymer coatings are used in touchscreens and medical devices.	Low-energy surfaces may be less effective at retaining antiviral agents.

**Table 3:** Advantages and challenges of the different materials and strategies for antiviral coatings.

Approach	Advantages	Challenges
**Metal-based coatings**	Broad-spectrum efficacy, durability	Potential toxicity, high cost
**Polymer-based coatings**	Versatility, metal-free	Limited long-term stability
**Photocatalytic coatings**	Light-activated, self-cleaning properties	Dependence on light exposure, wavelength and intensity
**Nanomaterial-Enhanced**	High efficacy, scalability	High cost, potential toxicity
**Bio-inspired coatings**	Eco-friendly, sustainable	Lower efficacy in some cases

**Table 4:** Comparison of Systems for Visible-light-activated Antiviral Coatings.

System	Mechanism	Advantages	Challenges	Applications
**Porphyrin-based coatings**	Generates singlet oxygen under visible light via photosensitization.	High efficiency; broad-spectrum activity; customizable.	Potential photobleaching; stability issues in some environments.	Medical textiles, air filters, hospital surfaces.
**Phthalocyanine-based coatings**	Produces singlet oxygen through photosensitization in the visible spectrum.	High photostability; strong visible light absorption.	Complex synthesis; cost of materials can be high.	Photodynamic therapy, antiviral paints.
**Carbon-based coatings**	Functionalized graphene or carbon dots generate ROS upon visible light activation.	High surface area; excellent stability; tunable properties.	Cost and complexity ofsynthesis and functionalization	High-performance antiviral surfaces, electronics.
**Metal oxide coatings**	Generates reactive oxygen species (ROS) like superoxide and hydroxyl radicals under light.	Durable; scalable; cost-effective.	Requires doping to activate under visible light.	Construction materials, water disinfection.
**Nanoparticle-Enhanced Coatings**	Embedded metal (e.g., Ag, Au) nanoparticles enhance light absorption and ROS production.	Synergistic effects; can be tuned for specific wavelengths.	High cost of noble metals; potential environmental concerns.	Medical devices, high-touch public surfaces.

**Table 5:** Design characteristics of antiviral coatings activated by light.

Characteristic	Description	Examples	Design Challenges
**Photosensitizer Material**	Generates reactive oxygen species (ROS) when exposed to light.	Porphyrins, Phthalocyanines, Carbon Dots, Metal-doped Semiconductors (TiO_2_, ZnO).	Stability under prolonged exposure, photobleaching risk.
**Light Activation Range**	Wavelength of light required for activation (UV, visible, or near-infrared).	Visible light (400–700 nm) preferred for safe, continuous activation.	Efficiency drops under low-intensity or short-duration lighting.
**ROS Production Efficiency**	Ability to generate singlet oxygen, hydroxyl radicals, or superoxide anions.	^1^O_2_ production via porphyrins; ^•^OH via TiO_2_ under UV light.	Maintaining high ROS yield while reducing energy requirements.
**Surface Porosity**	Controls the interaction of viral particles and storage of photosensitizers.	Porous membranes loaded with photosensitizers.	Excessive porosity may trap viruses, reducing cleaning efficiency.
**Hydrophobicity**	Minimizes viral attachment by repelling water and contaminants.	Superhydrophobic surfaces with self-cleaning properties.	Balancing water repellency with compatibility for ROS generation.
**Photostability**	Resistance to degradation under prolonged light exposure.	Stable materials like carbon dots or metal-doped semiconductors.	Ensuring longevity without compromising functionality.
**Surface Energy**	Influences adhesion of viruses and retention of antiviral agents.	High-energy surfaces for enhanced ROS production; low-energy surfaces to resist viral adhesion.	Precision engineering to balance antiviral efficacy and contamination resistance.
**Mechanical Durability**	Ability to withstand wear and maintain functionality over time.	Polymer-based coatings with integrated nanoparticles.	Durability under environmental stress (abrasion, humidity).
**Compatibility**	Adhesion to diverse substrates like metals, glass, or polymers.	Functionalized coatings for medical textiles, hospital surfaces, and air filters.	Tailoring deposition techniques (spray-coating, dip-coating) to various materials.
**Light Source Dependency**	Requirement for external or ambient light to sustain activation.	Ambient light in healthcare environments; UV in sterilization settings.	Reduced efficacy in poorly lit areas; requires hybrid approaches for continuous protection.
**Broad-Spectrum Activity**	Efficacy against a wide range of viruses and bacteria.	Effective against both enveloped (SARS-CoV-2) and non-enveloped (Norovirus) viruses.	Enhancing action against non-enveloped viruses, which are more resistant to ROS.
**Environmental Safety**	Minimizing harmful effects on the environment and human health.	Non-toxic materials like carbon-based systems; reduced use of heavy metals.	Balancing high efficacy with eco-friendliness and safe disposal.

**Table 6:** Comparison of antiviral efficacy of hydroxyl radicals and singlet oxygen.

Aspect	Hydroxyl Radicals (^•^OH)	Singlet Oxygen
**Reactivity**	Extremely reactive, non-selective	Highly selective, moderate reactivity
**Target Viruses**	Effective against both enveloped and non-enveloped viruses	More effective against enveloped viruses
**Action Mechanism**	Indiscriminately attacks lipids, proteins, and nucleic acids	Targets specific lipid bonds and amino acids
**Stability**	Very short-lived	Longer-lived
**Diffusion Distance**	Limited to immediate proximity	Greater range
**Generation Methods**	Fenton reaction, photocatalysis	Photosensitizers under visible light
**Applications**	Suitable for sterilization in high-concentration systems	Ideal for light-activated coatings and surfaces

## Data Availability

No new data were created or analyzed in this study.
